# Time-dependent diffusion MRI probes cerebellar microstructural alterations in a mouse model of Down syndrome

**DOI:** 10.1093/braincomms/fcab062

**Published:** 2021-04-05

**Authors:** Dan Wu, Yi Zhang, Bei Cheng, Susumu Mori, Roger H Reeves, Feng J Gao

**Affiliations:** 1 Key Laboratory for Biomedical Engineering of Ministry of Education, Department of Biomedical Engineering, College of Biomedical Engineering & Instrument Science, Zhejiang University, Hangzhou, Zhejiang 310027, China; 2 Department of Radiology, Johns Hopkins School of Medicine, Baltimore, MD 21205, USA; 3 Department of Physiology, Johns Hopkins School of Medicine, Baltimore, MD 21205, USA

**Keywords:** diffusion MRI, diffusion-time-dependence, oscillating gradient, cerebellar microstructure, Down syndrome

## Abstract

The cerebellum is a complex system with distinct cortical laminar organization. Alterations in cerebellar microstructure are common and associated with many factors such as genetics, cancer and ageing. Diffusion MRI (dMRI) provides a non-invasive tool to map the brain structural organization, and the recently proposed diffusion-time (*t_d_*)-dependent dMRI further improves its capability to probe the cellular and axonal/dendritic microstructures by measuring water diffusion at multiple spatial scales. The *t_d_*-dependent diffusion profile in the cerebellum and its utility in detecting cerebellar disorders, however, are not yet elucidated. Here, we first deciphered the spatial correspondence between dMRI contrast and cerebellar layers, based on which the cerebellar layer-specific *t_d_*-dependent dMRI patterns were characterized in both euploid and Ts65Dn mice, a mouse model of Down syndrome. Using oscillating gradient dMRI, which accesses diffusion at short *t_d_*’s by modulating the oscillating frequency, we detected subtle changes in the apparent diffusivity coefficient of the cerebellar internal granular layer and Purkinje cell layer of Ts65Dn mice that were not detectable by conventional pulsed gradient dMRI. The detection sensitivity of oscillating gradient dMRI increased with the oscillating frequency at both the neonatal and adult stages. The *t_d_*-dependence, quantified by ΔADC map, was reduced in Ts65Dn mice, likely associated with the reduced granule cell density and abnormal dendritic arborization of Purkinje cells as revealed from histological evidence. Our study demonstrates superior sensitivity of short-*t_d_* diffusion using oscillating gradient dMRI to detect cerebellar microstructural changes in Down syndrome, suggesting the potential application of this technique in cerebellar disorders.

## Teaser

Cerebellar hypoplasia is common in people with Down syndrome. Wu et al. decipher the spatial correspondence between cerebellar layers and diffusion MRI contrasts. The layer-specific oscillating gradient diffusion MRI at short diffusion-time shows superior sensitivity to detect microstructure alterations in both developing and mature cerebellar layers of a Down syndrome mouse model.

## Graphical Abstract

**Graphical Abstract:**
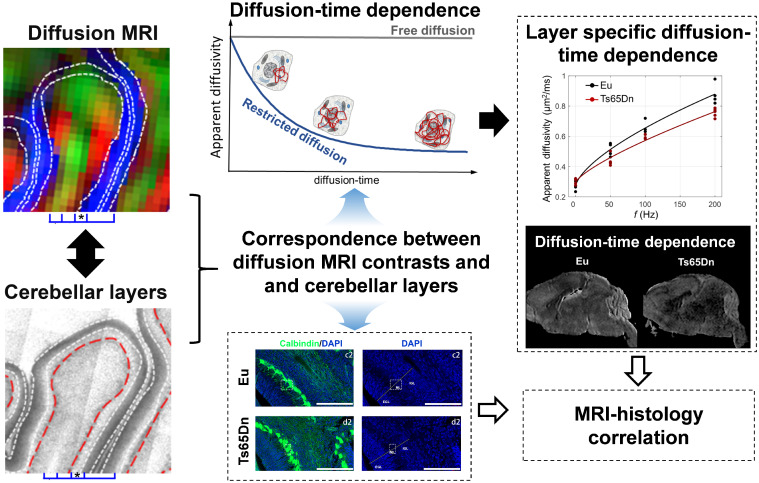

## Introduction

The mammalian cerebellum, consisting of complex foliation, is responsible for not only motor coordination^1^ but also high-order cognitive functions.^2^ Cerebellar diseases due to genetic dysfunctions are common during brain development.^3^ One of the first steps of studying cerebellar disorders is to characterize the phenotypes, and histology has been the tool of choice. However, histological approaches are known to have a limited scope of views, e.g. the examination is limited to the locations, thickness and orientations of the given slices. MRI overcomes the limitation by offering a three-dimensional (3D) virtual dissection of the tissue. On the other hand, compared to histology that has almost unlimited number of staining methods, MRI can only offer a handful of contrast mechanisms that mostly reflect macroscopic information of the brain, e.g. white matter versus gray matter. The development of new MRI contrasts targeting cellular and subcellular information could significantly expand its applications in both basic and clinical research.

**Figure 1:**
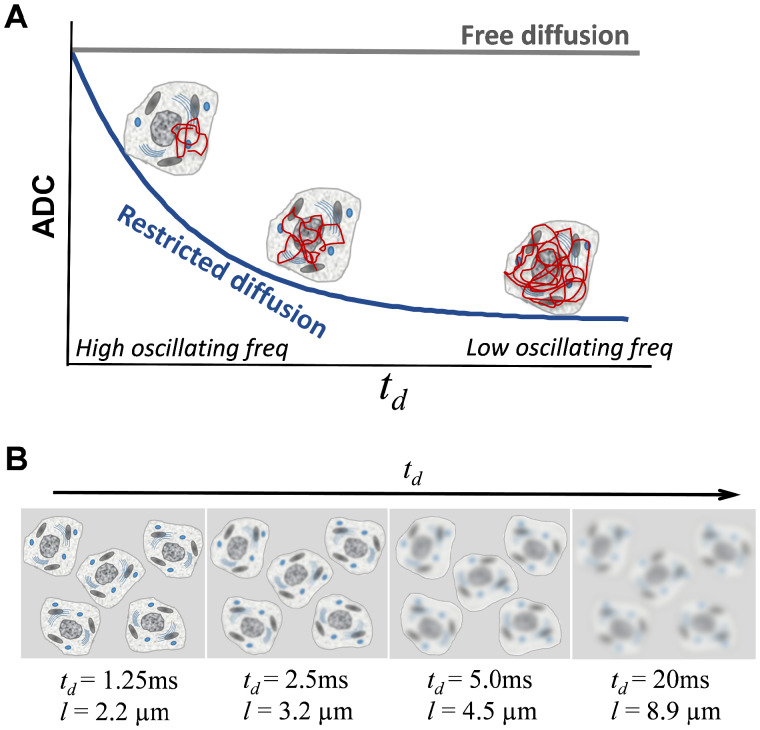
**Schematics of the principle of *t_d_-*dependent dMRI.** (**A**) *t_d_-*dependent ADC changes. ADC measured in the tissue microstructures decreases as *t_d_* increases or oscillating frequency *f* decreases, because at short *t_d_*, the diffusion distance (*l*) of water molecules is short and the diffusion trajectory is restricted by only a few subcellular organelles in its immediate neighbourhood; while at long *t_d_*, *l* is long and the diffusion process becomes more restricted by multiple structural barriers, such as the nuclei and cell membrane. (**B**) Concept of ‘coarse-graining’.^45^ At short *t_d_*, water diffusion is sensitive to small structures at the scale of diffusion distance *l*. As *t_d_* increases, *l* increases and details of the small cellular structures becomes blurred to the diffusing water molecules.

Diffusion MRI (dMRI) is one of the few non-invasive imaging approaches to probe brain structural information on a microscopic scale. Measuring dMRI signals with various diffusion gradient encoding schemes enables inferences about tissue microstructural composition and characterization of pathological changes. Tremendous progress has been made to reconstruct the white matter tracts or orientation information of tissue organization by exploring the direction and strength of diffusion encoding gradients, e.g. based on diffusion tensor imaging (DTI)^4^ or high angular-resolution diffusion imaging.^5^ Recently, the diffusion-time (*t_d_*)-dependent dMRI technique has demonstrated unique advantages in depicting tissue microstructural information^6–8^ beyond the directional information. The *t_d_*, a critical parameter in diffusion encoding, sets the measuring window for the water molecules to interact with the microscopic surroundings and dictates the sensitivity of dMRI signals to microstructures of different spatial scales (Fig. 1). Due to the gradient strength limitations, the achievable *t_d_* is limited to >5 ms on preclinical scanners and >10 ms on clinical systems using the conventional pulsed gradient (PG).^9^ The development of oscillating gradient (OG) has enabled us to access diffusion at shorter *t_d_* than PG by repeating sine or cosine shaped gradients, thereby sensitizing the diffusion to structures at small spatial scales.^8^

The dMRI signals obtained at varying *t_d_*’s give rise to distinct tissue contrasts in the brain. Aggarwal et al.^10^ demonstrate in mice that the apparent diffusivity coefficient (ADC) map of OG-dMRI at the high oscillating frequency (short *t_d_*) highlights the neuronal structures with densely packed cells, e.g. dentate gyrus of the hippocampus and granular layer of the cerebellum. Colvin et al.^11^ report that ADC map at high oscillating frequency shows an increased contrast and spatial heterogeneity in glioblastomas of the rat brain and detects early intracellular changes in glioblastomas after drug treatment, prior to the tissue cellularity changes that can be detected with conventional MRI.^12^ Bongers et al.^13^ suggest that OG-dMRI provides higher sensitivity than PG-dMRI in detecting radiation response in glioblastoma. We recently demonstrated in a mouse model of neonatal hypoxia-ischaemia that OG-dMRI enhances the sensitivity to detect early and subtle ischaemic injury in the hippocampus.^14^ In combination with compartmentalized biophysical models,^15–17^ the *t_d_*-dependence of water diffusion has been used to quantify microstructural properties, such as the surface-to-volume ratio, cell size, axonal diameter, intra-cellular fraction and nucleus-to-cell ratio.^15–22^

Given the unique sensitivity of *t_d_*-dependent dMRI, we speculate that it would be useful for characterizing complex microstructures in the cerebellum. Each lobe of the cerebellar cortex is organized into a layered architecture consisting of the Purkinje cell (PC) layer (PCL), granule cell layer (GCL) and molecular layer (ML). Those neurons are connected within the cerebellum and to other brain regions through several pathways, including the climbing fibres that ascend to PCs, mossy fibres that innervate GCs, and parallel fibres that connect interneurons in the ML.^23^ The dMRI-based metrics exhibit rich contrasts in delineating the cerebellar microstructure, such as the layered architecture^24^^,^^25^ and cerebellar circuitry.^26^^,^^27^ However, the spatial correspondence between dMRI contrast and the anatomical organization is not straightforward, especially in the context of cerebellar development during the postnatal period.^28^ Interestingly, compared to other brain structures, the cerebellum exhibits the highest *t_d_*-dependence,^10^^,^^29^^,^^30^ possibly related to the large population of densely packed granule cells (GCs).^31^ However, the *t_d_*-dependent diffusion profiles in different cerebellar layers and the change of *t_d_*-dependence with cerebellar development or pathology remain largely unknown.

Down syndrome (DS), which is caused by trisomy of human chromosome 21 (HSA21), occurs in about 1 in every 800 new births.^32^ HSA21 has >200 protein-coding genes and >600 non-protein-coding genes, and trisomy of such a large genetic content can cause genome-wide expression imbalance in every cell and potentially disrupt homeostasis in all body systems.^33^ Individuals with DS have common neurological phenotypes including intellectual disability and early-onset dementia,^34^^,^^35^ and also show smaller brain and disproportionately small cerebellum in conventional MRI.^36^^,^^37^ Ts65Dn, the first viable and most widely used DS mouse model, is segmentally trisomic for about 92 of 160 HSA21 mouse orthologs (not including keratin-associated protein genes) and exhibits DS phenotypes including cognitive impairment and disproportionately small cerebellum.^38–41^ Cellular pathology in Ts65Dn cerebellum such as impaired GC precursor proliferation, reduced GC density, and PC deficits has been reported.^31^^,^^42–44^

Here, we first mapped spatial correspondence between different cerebellar layers and dMRI contrasts in both developing and adult mice. Then based on the spatial correspondence, we compared the sensitivity of PG-dMRI and OG-dMRI at a range of oscillating frequencies with a high-resolution 3D gradient spin-echo sequence to detect cerebellar microstructural alterations in both developing and adult Ts65Dn mice and provided corresponding histological evidence. Our results demonstrate that *t_d_*-dependence dMRI, especially diffusion at short *t_d_*, has superior sensitivity of detecting cerebellar abnormalities associated with DS. The technique is translatable and potentially paves a much-needed path to the precision neuroimaging of cerebellar and neurological disorders.

## Methods

### Animal research

This study was carried out in accordance with the recommendations of the NIH Guide for the Care and Use of Laboratory Animals and the Johns Hopkins University (JHU) Institute of Animal Care and Use Committee. The protocol was approved by the JHU Institute of Animal Care and Use Committee. Mice were maintained in a JHU animal facility with 14-h light/10-h dark cycle, temperatures of 65–75°F (∼18–23°C) with 40–60% humidity, and fed with standard chow and in-cage automatic water. We followed the ARRIVE guidance (https://arriveguidelines.org/) to report all animal research.

Ts65Dn line is maintained on the C57BL/6J × C3H/HeJ (B6C3H, JAX 100010) background though both female transmission [B6C3H-Ts65Dn females × B6C3H euploid (Eu) males] and male transmission (B6C3H-Ts65Dn males × B6C3H Eu females), and young adult B6C3F1 males from The Jackson Laboratory (JAX 100010) are imported into the colony every 6 months to refresh breeders to prevent genetic drift. In this study, B6C3H-Ts65Dn males were mating with B6C3H Eu females to generate five pairs of postnatal day (P)6 Ts65Dn and Eu littermates (both males and females used) and six pairs of P31 Ts65Dn and Eu littermates (all males) for MRI and histological analysis. Gli1^tm2Alj^/J mice (Gli1^LacZ/+^, JAX 008211) have been backcrossed into C57BL/6J (B6) background for more than 6 generations in the lab and are maintained on the B6 genetic background. In this study, B6-Gli1^LacZ/+^ female mice were mating with B6C3H-Ts65Dn male mice to generate two pairs of P6 Ts65Dn; Gli1^LacZ/+^ and Eu; Gli1^LacZ/+^ littermates that were used for the X-gal and anti-Calbindin co-staining, and the X-gal staining and dMRI contrast images of Eu; Gli1^LacZ/+^ cerebellum was used to generate the spatial correspondence analysis. For genotyping, mouse tail samples were digested in a 1.5 ml tube containing 600 µl of lysis buffer (50 mM Tris pH8, 100 mM EDTA, 0.5% SDS, and 400 mM NaCl) plus 15 µl of 20 mg/ml Proteinase K at 55°C overnight. The tube was added with 180 µl the saturated NaCl solution, which was mixed well and centrifuged at 13 000 rpm for 10 min at 4°C. The supernatant was transferred to another 1.5 ml tube and mixed with 700 µl 100% EtOH to precipitate DNA, followed by centrifugation at 13 000 rpm 4°C for 10 min. The DNA pellet was dried and resuspended in 500 µl DEPC treated H_2_O. For Ts65Dn genotyping, the trisomic primer set ‘C17F (GTGGCAAGAGACTCAAATTCAAC) and C16R (TGGCTTATTATTATCAGGGCATTT)’ and the internal control primer set ‘IMR5 (AAAGTCGCTCTGAGTTGTT) and IMR6 (GGAGCGGGAGAAATGGATA)’ were used. For Gli1^LacZ/+^ genotyping, the primer set ‘Common F (GGGATCTGTGCCTGAAACTG), Mutant R (TCTGCCAGTTTGAGGGGACGAC), and WT R (AGGTGAGACGACTGCCAAGT)’ were used. The Touchdown PCR cycle (Supplementary Table 1) was used to amplify PCR products, which were analysed in 2% agarose gel.

For *ex vivo* brain MRI, mice were anaesthetized with isoflurane and transcardially perfused with PBS and then 4% paraformaldehyde (PFA) in PBS buffer. The mouse heads were preserved in 4% PFA for a week, and then kept in PBS solution with 0.1 mM gadopentetate dimeglumine (Magnevist, Berlex Imaging, Wayne, NJ, USA) at 4°C for another week before the MRI scan.

### Principle of *t_d_*-dependent dMRI

*t_d_*-dependent dMRI probes the tissue microstructure by measuring water diffusion at varying *t_d_*’s. Unlike ADC in free water that does not change with *t_d_*, ADC of restricted diffusion of water molecules that are confined by the microstructural boundaries decreases as *t_d_* increases or oscillating frequency *f* decreases (*t_d_*∝1/*f*) (Fig. 1A). This is because at short *t_d_*, the diffusion distance is short, and thus, water diffusion is only restricted by few obstacles such as the subcellular organelles in its immediate neighbourhood; whereas as *t_d_* increases, the water molecules travel a long distance across multiple structural barriers, such as the nuclei and cell membrane, and thus, become more restricted. Therefore, water diffusion is sensitized to structures close to the scale of diffusion distance $l=\sqrt{2Dt_{d}}$, where *D* is the free diffusion coefficient. At very short *t_d_*, *l* is short, e.g. *l* = 2.2 µm for *t_d_* at 1.25 ms (corresponding to *f* = 200 Hz), and the water molecules can effectively probe the details of the microstructural environment (Fig. 1B). As *t_d_* increases, *l* increases, e.g. *l* = 8.9 µm for *t_d_* at 20 ms, which exceeds the size of the subcellular organelles or cells, and thus, and the diffusivity measurements reflect both large and small scale structural features at long *t_d_*, as the so-called ‘coarse-graining’^45^ takes place.

### Data acquisition

Postmortem mouse heads were scanned on an 11.7T Bruker vertical-bore NMR spectrometer (Bruker BioSpin, Billerica, MA, USA) with a Micro2.5 gradient system (maximum gradient strength of 1500 mT/m) and a 15 mm diameter birdcage transceiver RF coil. The specimens were immersed in fomblin (Fomblin Perfluoropolyether, Solvay Solexis, Thorofare, NJ, USA) during the scan, and the temperature was maintained at 22°C by the spectrometer’s temperature control system.

dMRI data were acquired using a house-made 3D diffusion-weighted gradient spin-echo sequence^46^ with oscillating or pulsed gradients. The 3D images were acquired in sagittal orientation with frequency-encoding along the rostral-caudal axis, the first phase-encoding using echo-planar imaging readout along the anterior-posterior axis, and the second phase-encoding using turbo-spin echo readout along the left-right axis. The following parameters were used: echo time/repetition time = 63.8/800 ms, two signal averages, turbo-spin echo factor of four, echo-planar imaging factor of five, isotropic resolution of 0.08 mm for the P6 brains and 0.1 mm for the P31 brains. OG-dMRI was performed at *f* of 50 Hz, 100 Hz and 200 Hz with 1, 2 and 4 oscillating cycles, respectively, *b*-values of 1 ms/µm^2^ in 10 diffusion directions, and two non-diffusion weighted images (b_0_). PG-dMRI was acquired with diffusion duration (δ)/diffusion separation (Δ) = 5/20 ms, and the other parameters matched to the OG-dMRI scans. The effective *t_d_* was approximately $\frac{1}{4f}$,^47^ which was 5, 2.5 and 1.25 ms for *f* of 50, 100 and 200 Hz, respectively, and the effective *t_d_* of PG-dMRI was approximated by Δ-δ/3 = 18.3 ms. DTI data were acquired to assist the definition of cerebellar layers, using the diffusion-weighted gradient spin-echo sequence with echo time/repetition time = 33.5/700 ms, δ/Δ = 3.4/8.5 ms, *b*-value = 2 ms/µm^2^ for the P6 mice and 2.8 ms/µm^2^ for the P31 mice, 30 diffusion directions, three b_0_ images and resolution and geometry matched to the PG- and OG-dMRI scans.

### Data analysis

The images acquired by the 3D GRASE sequence were zero-padded to 0.4 mm and 0.5 mm isotropic resolution for the P6 and P31 mouse brains, respectively. Mean ADC maps were generated at each *t_d_* according to *S* = *S*_0_$\cdot e^{-b\cdot D}$, where *S* and *S*_0_ are the diffusion-weighted and non-weighted signals. DTI reconstruction was performed in DtiStudio (www.mristudio.org) to obtain the directional encoded colourmap (DEC) with the colours encoded for the direction of well-organized tissue microstructures that are coherently aligned towards certain orientation.

**Figure 2:**
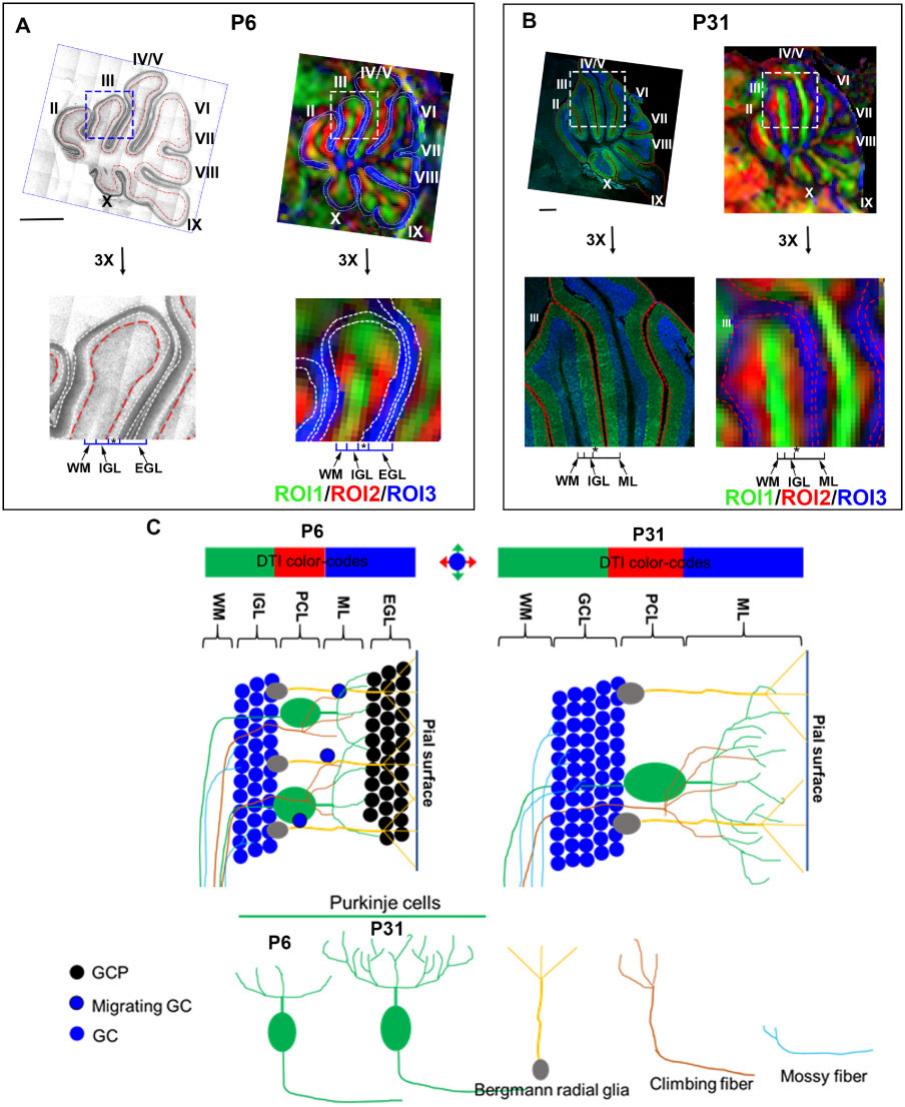
**Spatial correspondence between the cerebellar layers and DTI contrasts.** For the centre part of the cerebellum (lobule III and lobule IV/V) that are positioned in a superior-inferior orientation, the DTI-based direction encoded colourmap (DEC) is coloured in green (ROI1), and the inner rim is coloured in red (ROI2), and the outer rim is coloured in the blue (ROI3) in both the P6 (**A**) and P31 (**B**) brains. (**A**) X-gal staining and the corresponding DEC images of cerebellum of P6 Gli1^LacZ/+^ mice (top) and 3× enlargement of lobule III (bottom). Two types of Gli1-positive cells in the P6 cerebellum, external granule cells (between two white dash lines) that label external GC layer (EGL) and Bergmann glial cells (red dash lines) that separate the internal granule layer (IGL) and PC layer (PCL), define the different cerebellar layers of WM, IGL, PCL (labelled by *) and EGL. The relative position of each layer is inferred in DEC images. (**B**) Anti-Calbindin immunostaining and the corresponding DEC images of the P31 cerebellum (top panels) and 3× enlargement of lobule III (bottom panels). The sections are immunostained with anti-calbindin (green) and DAPI (blue) for visualization of the different cerebellar layers, and the relative position of each layer is inferred on DEC images. (**C**) Schematics to decipher DTI-based colour-codes into the biophysical layers of P6 and P31 mouse cerebellum. The ascending WM that runs in the superior-inferior direction gives rise to the green colour in the DEC images; the red colour mainly comes from PC proximal dendrites, the branches of the climbing fibres that connect to the PCs, the mossy fibres that connect to GCs, and the radial fibres of Bergmann glia, which run perpendicular to the surface of the lobules; and the blue colour is possibly driven by parallel fibres, dendritic branches of PCs, and terminal radial fibres of Bergmann glia in ML that run perpendicular to the sagittal plane. See Supplementary Fig. 5 for separate channel images of A and B.

The mouse brains were automatically segmented into 58 regions of interest (ROIs) (Supplementary Fig. 1) based on a pre-defined DTI atlas of the developing mouse brain^48^ by registering the individual brains to the atlas brain and then back-transforming the ROI definitions to the subject space.^46^ The cerebellar ROI was then manually corrected to obtain the cerebellar volume and average DTI metrics. We then manually delineated the cerebellar layers according to the tissue orientational information based on DEC, namely, green, red and blue coloured regions (ROI 1–3) in lobule III and lobule IV and V on the mid-sagittal section (Fig. 2).

Linear regression between the ROI-averaged ADC values at multiple *f* (*f* = 0 for PG-dMRI) and $\sqrt{f}$ was performed, as the diffusivity in the short *t_d_* regime has shown to have a linear relationship with $\sqrt{f}$.^18^^,^^49^^,^^50^ The slope of the linear regression (ΔADC) was used to quantify the degree of *t_d_*-dependence:

(1) $$\text{ADC}\left(f\right)=\Delta \text{ADC}\sqrt{f}+{\text{ADC}}_{0}$$

In addition, since the linear relationship does not necessarily hold for all *f* ranges,^49^ we also examined the power-law relationship^49^ between ADC(*f*) and *f* according to

(2) $$\text{ADC}\left(f\right)=\alpha f^{\theta }+{\;\text{ADC}}_{0}$$

where ADC_0_ is the diffusivity at zero frequency; *α* is a scaling factor related to the degree of *t_d_*-dependence; the exponent *θ* is the diffusion dispersion rate reflecting the structural disorder.^49^ Fitting of equations (1) and (2) was performed in MATLAB R2018a (Mathworks, Inc., Natick, MA, USA), and the related script is available at (https://www.mathworks.com/matlabcentral/fileexchange/86307-tdmri_curvefit).

### X-gal staining (also known as LacZ staining)

Gli1^LacZ/+^ mice were transcardially perfused with PBS and 4% PFA. The isolated brains were fixed in 4% PFA at 4°C overnight, transferred into 30% sucrose at 4°C for 48 h, and embedded into optimal cutting temperature compound. The 40 µm sagittal cryosections were collected using Leica Cryostat CM 3050S and mounted on the glass slides, which were dried with natural air at room temperature. The fixation buffer (2% paraformaldehyde, 0.02% glutaraldehyde, 2 mM MgCl2 in PBS) was used to fix slides for 10 min at room temperature, followed by a 10 min PBS wash and another 10 min wash using wash buffer (5 mM EGTA, 0.01% Deoxycholate, 0.02% NP40, 2 mM MgCl_2_ in 0.1 M phosphate buffer). The slides were then incubating with X-gal staining solution [5 mM K3Fe(CN)6, 5 mM K4Fe(CN)6, 5 mM EGTA, 0.01% Deoxycholate, 0.02% NP40, 2 mM MgC12, 1 mg/ml X-gal] at 37°C in the dark overnight, followed by a wash in PBS for 10 min and a wash in distilled water for 5 min. X-gal stained slides (Fig. 2A and Supplementary Fig. 2) were co-stained with anti-Calbindin antibodies for confocal imaging (Zeiss LSM800 GaAsP, MicFac).

### Immunostaining

The mouse brain preparation prior to the cryo-sectioning was the same as that for X-gal staining. Slides of 40 µm sagittal cryosections with or without X-gal staining were post-fixed in 4% PFA for 30 min and rinsed twice with PBS, which were then permeabilized and blocked with blocking buffer (0.5% triton X-100 in PBS with 10% goat serum) for 1 h. The slides were probed with primary antibodies (anti-Calbindin to label the PCs, Cell Signaling #13176) diluted in blocking buffer at 4°C overnight (negative control was not treated with primary antibodies), followed by three times of 10 min PBST-T (0.1% Tween-20 in PBS) wash, which were then probed with Alexa Fluor conjugated secondary antibodies diluted in blocking buffer for 1 h, followed by three washes in PBS-T, 10 min each. The slides were stained with DAPI [1 µl DAPI (MilliporeSigma #10236276001) diluted in 50 ml PBS-T] for 10 min and sealed with coverslips using ‘ProLong Gold Antifade Mountant with DAPI’. The Slides were dried with nature air at room temperature in dark overnight and imaged using Zeiss LSM800 GaAsP (MicFac). Z-stack and tile scan confocal images were stitched in Zen (Zeiss). To compare the linear length of cerebellar layers (Calbindin^+^, IGL, ML plus EGL of P6, and Calbindin^+^ and GCL of P31) between Eu and Ts65Dn, the same cerebellar folia (lobule III, IV and V) of the mid-sagittal surface from both Eu and Ts65Dn were measured in Fiji (Image J), and the average number of the lobule III-IV of each animal was compared.

### Monte Carlo simulation

Monte Carlo simulation was performed to examine the *t_d_*-dependent ADC changes with the change of microstructural size and fraction, using the Camino toolbox (http://camino.cs.ucl.ac.uk/). Cellular phantoms were composed of impermeable spheres substrates with square packing, diameters varying from 4 to 18 µm, and intracellular fractions varying from 0.06 (separation = 2*diameter) to 0.39 (separation = 1.1*diameter) for each diameter. Neurite phantoms were composed of cylinder substrates with square packing, diameters varying from 0.4 to 1.8 µm, and intra-neurite fractions varying from 0.35 (separation = 1.5*diameter) to 0.69 (separation = 1.1*diameter) for each diameter. PG and OG gradients were simulated at Δ = 20 ms and *f *=* *50, 100 and 200 Hz, similar to the animal experiment settings.

### Statistical analysis

All statistical tests were performed using Prism Graphpad (https://www.graphpad.com/scientific-software/prism/). The ADC differences between Eu and Ts65Dn groups at four oscillating frequencies (0, 50, 100, 200 Hz) were compared by two-way analysis of variance with the groups being one factor and the frequencies being the other factor, followed by *posthoc t*-tests between the groups at each frequency. ΔADC values were compared between Eu and Ts65Dn groups in the three cerebellar ROIs by two-way analysis of variance with two factors of the groups and ROIs, followed by *posthoc t*-tests between the groups in each ROI. The relative thickness of cerebellar layers (Ts65Dn/Eu) was analysed by one-way analysis of variance and Dunnett’s multiple comparison test. Data were represented as mean ± SEM. All significance thresholds were set at *P* < 0.05 unless otherwise stated. The statistical methods and sample size were also shown in the figure legend. The detailed statistical analysis for each part of the results is also available in Supplementary Table 2.

### Data availability

The key part of the MRI calculation is related to the linear model in Equation (1) and the power-law relationship in Equation (2), and corresponding Matlab scripts are available on Matlab Central (https://www.mathworks.com/matlabcentral/fileexchange/86307-tdmri_curvefit). The other data are available in the paper.

## Results

### Volumetric and DTI characteristics of the Ts65Dn cerebellum

We first examined the overall volumetric and DTI changes of the entire cerebellum of the Ts65Dn mice (Table 1). At P6, the whole-brain volume and cerebellar volume of the Ts65Dn mice were 13% and 34% smaller than Eu mice, respectively (*P* <* *0.001), and the cerebellum-to-brain ratio was also significantly smaller than Eu (*P* <* *0.01). At P31, the volumetric difference was smaller but still significant, with whole-brain volume and cerebellar volume of Ts65Dn 7% (*P* =* *0.042) and 14% (*P* =* *0.002) smaller than those of Eu, respectively. The cerebellum-to-brain ratio was also smaller at P31 (*P* =* *0.01). DTI-based fractional anisotropy and ADC measurements over the entire cerebellum were similar between the two groups at P6 or P31, indicating a necessity of layer-specific analysis of the dMRI measurements in the cerebellum.

**Table 1:** Volumetric and DTI measurements of the entire cerebellum in the Ts65Dn and Eu mice at P6 and P31

	P6		P31	
	Eu (*n *=* *5)	Ts65Dn (*n *=* *5)	Eu (*n *=* *6)	Ts65Dn (*n *=* *6)
Whole brain volume (mm^3^)	408.09 ± 14.92	355.52 ± 23.26***	507.45 ± 21.20	475.83 ± 19.19^*^
Cerebellar volume (mm^3^)	24.95 ± 3.20	16.48 ± 2.76***	64.21 ± 3.18	55.26 ± 5.26^**^
Cerebellar volume ratio (%)	6.11 ± 0.73	4.63 ± 0.67^**^	12.65 ± 0.36	11.60 ± 0.82^**^
Cerebellar FA	0.22 ± 0.02	0.24 ± 0.02	0.25 ± 0.016	0.26 ± 0.015
Cerebellar ADC (µm^2^/ms)	0.32 ± 0.01	0.33 ± 0.01	0.26 ± 0.01	0.27 ± 0.01

* *P *<* *0.05,

** *P *<* *0.01,

** *P *<* *0.001 using two-tailed *t*-test with unequal variance.

### Spatial correspondence between dMRI contrast and cerebellar layers

Since the mouse cerebellar cortex consists of laminar structures in each lobule and each layer has distinct microstructural composition and function, it is essential to perform layer-specific analysis. This task, however, is not straightforward given the indirect correspondence between the DTI contrast and biological laminar definition that was not addressed previously. DTI-based DEC images of cerebellum lobules exhibited a three-layer organization at both P6 and P31 (Fig. 2A and B). We compared the DTI patterns of the neonatal (P6) and adult (P31) mouse cerebellum with the histological sections in detail.

As X-gal staining of Gli1^LacZ/+^ cerebellum provides the extra layer information such as external granular layer (EGL) for P6 but not adult mice,^51^ to distinguish cerebellar layers of P6 mice, brain sections of Eu Gli1^LacZ/+^ mice were co-stained with X-gal and anti-Calbindin/DAPI (Supplementary Fig. 2A). The comparison of cerebellar layer boundaries from single-channel images [LacZ (ESID transmitted light), DAPI (blue), Calbindin (green)] and the merger of them (LacZ/Calbindin/DAPI) showed that the LacZ channel alone was sufficient to delineate the four distinct cerebellar layers [white matter (WM), internal granular layer (IGL), PCL and EGL] and had reduced visual complexity for layer definition compared to the merger (Supplementary Fig. 2B). Therefore, we used the LacZ single-channel images rather than the merger images to compare with cerebellar DTI contrasts to generate the spatial correspondence at P6 (Fig. 2A). Eu P31 mouse cerebellum was immunostained with anti-Calbindin/DAPI, and the dual staining was sufficient to show four layers including WM, GCL, PCL and ML (Fig. 2B). According to the relative position, proportion, and the microstructural composition in each layer, it can be inferred that ROI1, coloured in green on the DEC images, covers the WM and the inner part of IGL; ROI2, coloured in red on DEC, corresponds to the outer part of IGL and PCL; and ROI3, coloured in blue on DEC, points to EGL at P6 or ML at P31.

By incorporating previous extensive studies in the mouse cerebellar architecture and neuronal circuits,^52^ we generated a schematic model to decipher DTI-based colour-codes into the biophysical layers for both P6 and P31 cerebellum (Fig. 2C). The green colour on the DEC images is likely dominated by the ascending WM tracts that run in the superior-inferior direction. The red colour is mostly driven by the innervating branches of mossy fibres that connect to the GCs, the climbing fibres that connect to PCs, and PC proximal dendrites, which run perpendicular to the surface of the lobules. The outer rim coloured in blue could be associated with the parallel fibres, dendritic branches of PCs, and terminal radial fibres of Bergmann glia in ML that run perpendicular to the sagittal plane. Since the GCs in GCL of P31 (or IGL of P6) are nearly isotopically packed, colour-code in GCL/IGL is possibly driven by the neurites that run through it; and based on the proportions of the adjacent layers, GCL is likely to be included in both the green and red coloured ROIs.

### *t_d_*-dependent dMRI of the neonatal Ts65Dn cerebellum

**Figure 3:**
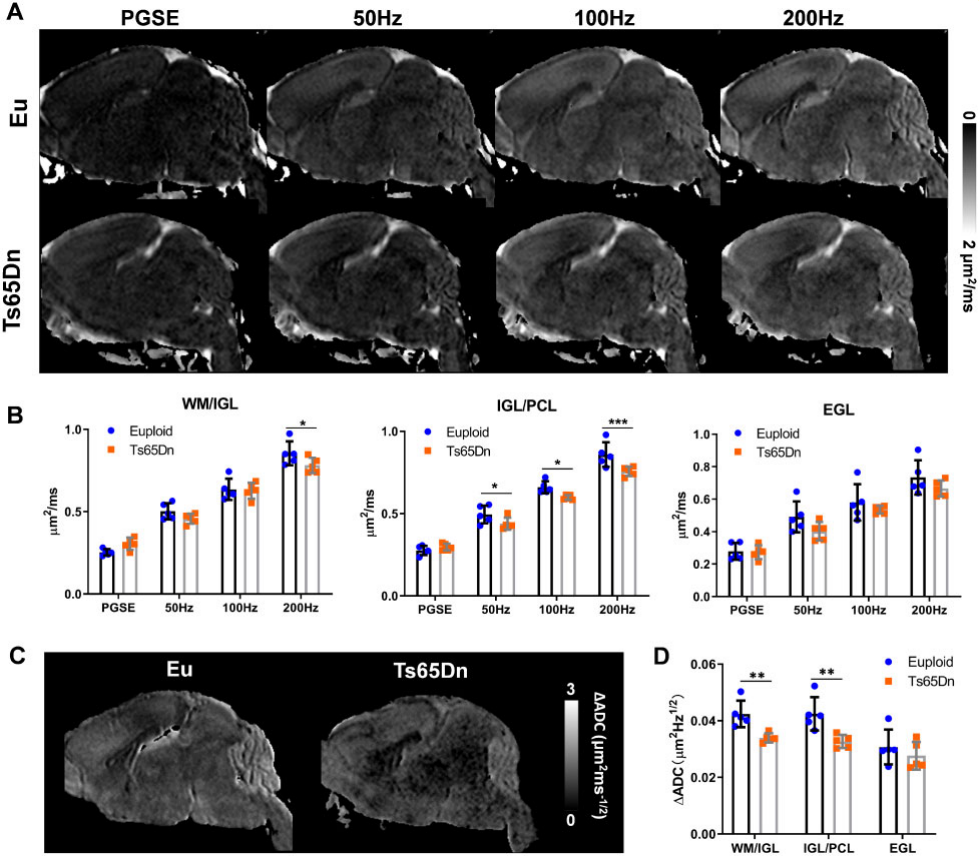
***T_d_*-dependent dMRI in the Eu and Ts65Dn mouse brains at P6.** (**A**) Mid-sagittal ADC maps of Eu and Ts65Dn mouse brains acquired with PG-dMRI and OG-dMRI at *f* of 50 Hz, 100 Hz and 200 Hz. (**B**) Comparison of ADC values between the Eu and Ts65Dn groups (*n *=* *5) using PG- and OG-dMRI scans in the cerebellar WM/IGL (ROI1), IGL/PCL (ROI2), EGL (ROI3). (**C**) Mid-sagittal ΔADC maps of the Eu and Ts65Dn mouse brains at P6. (**D**) Comparison of ΔADC between Eu and Ts65Dn groups in the three cerebellar ROIs. Data of (B) and (D) were analysed by two-way ANOVA and followed by *post hoc t*-test (*P <* *0.05, **P <* *0.01 and ***P <* *0.001), and see Supplementary Table 2 for detailed statistical analysis.

We compared P6 Eu and Ts65Dn brains to assess the sensitivity of PG-dMRI and OG-dMRI to detect abnormalities in the developing cerebellum. ADC maps of the P6 mouse brains exhibited marked *t_d_*-dependence with increasing ADC as *t_d_* decreased (or *f* increased), and the cerebellum showed the most prominent *t_d_*-dependence compared to the other brain structures (Fig. 3A). Unlike, PG-dMRI-based ADC (PG-ADC) that showed no significant difference in any of the three cerebellar ROIs between Eu and Ts65Dn (*P* >* *0.05), the OG-dMRI based ADC (OG-ADC) of Ts65Dn was significantly reduced in both ROI1 (WM/IGL) and ROI2 (IGL/PCL), but not in ROI3 (ML/EGL) (Fig. 3B). Particularity, OG-ADC in the IGL/PCL began to show group difference at *f* of 50 Hz (*P* =* *0.027), and the effect size continued to increase as *f* increased (*P* <* *0.001 at 200 Hz). The group difference in the WM/IGL was only detectable at *f* of 200 Hz (*P* =* *0.021). We calculated ΔADC according to Equation (1) to quantify the *t_d_*-dependence (Supplementary Fig. 3). The ΔADC map showed a visible reduction in the Ts65Dn cerebellum comparing to that of Eu (Fig. 3C), and the difference was more obvious than those in the raw ADC maps in Fig. 3A. The statistical analysis suggested a significant ΔADC reduction in both WM/IGL (*P* <* *0.01) and IGL/PCL (*P* <* *0.01) of Ts65Dn (Fig. 3D). The power-law relationship between ADC and *f* was also examined (Supplementary Fig. 4), and the corresponding results showed the *t_d_*-dependency, characterized by *α* in Equation (2), was lower in the Ts65Dn group, confirmatory to the ΔADC findings. Since the power-law fitting was not stable for individual analysis using only four frequencies to fit three unknown parameters in a non-linear relationship, we performed the fitting using all samples in each group (*n *=* *5 for P6 and *n *=* *6 for P31) instead, and therefore, statistical analysis was not available.

**Figure 4:**
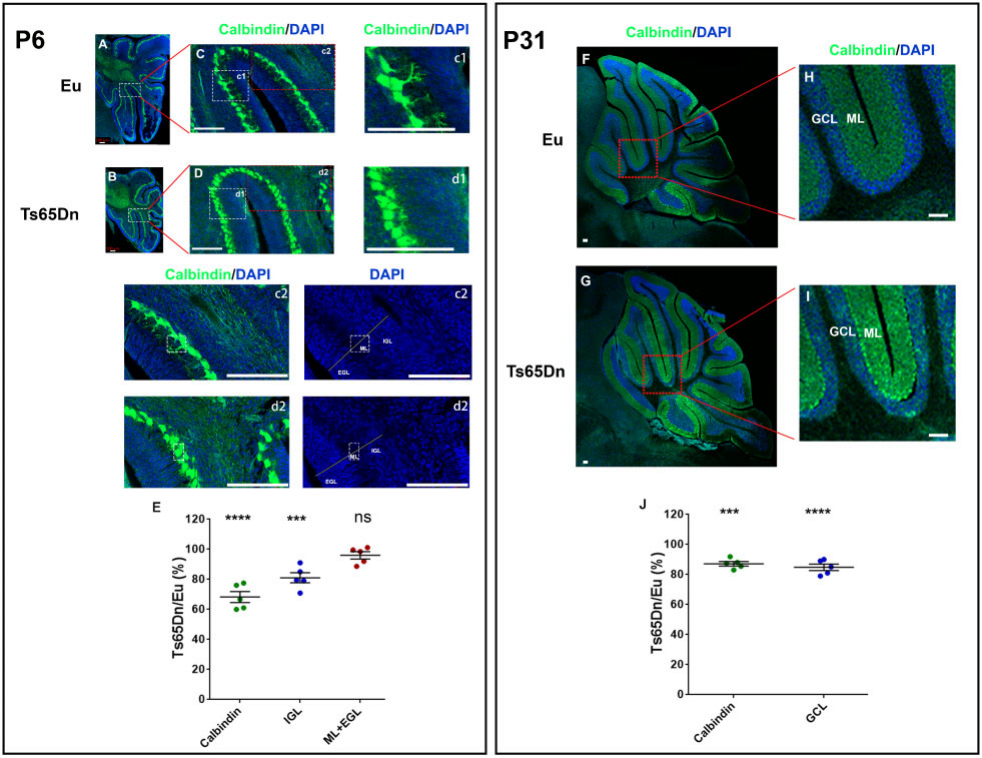
**Immunostaining of the PCs in Eu and Ts65Dn mouse brains at P6 (left panel) and P31 (right panel).** (**A** and **B**) Mid-sagittal brain sections from P6 Eu and Ts65Dn immunostained with anti-Calbindin (green) and counter-stained with DAPI (blue), and tile scan confocal images of the cerebellum are shown. (**C** and **D**) The enlargement of areas in A and B. The further enlargements of c1 and d1 to compare Purkinje cells morphology, and c2 and d2 enlargements to compare layers of cerebellar cortex including IGL, EGL, and ML. (**E**) The quantification of the thickness of cerebellar layers in Ts65Dn relative to Eu at P6. The Ts65Dn/Eu ratio of Calbindin-positive layer (the PC length, cell body plus dendrite), ‘IGL’, and ‘ML+EGL’ (PC dendrite plus EGL) are compared (*n = 5*). (**F** and **G**) Mid-sagittal brain sections from P31 Eu and Ts65Dn immunostained with anti-Calbindin (green) and counter-stained with DAPI (blue). (**H** and **I**) The enlargement of areas in F and G for comparing adult cerebellar cortex, including GCL and ML. (**J**) The quantification of the thickness of cerebellar layers in Ts65Dn relative to Eu at P31. The Ts65Dn/Eu ratio of Calbindin-positive layer (the PC length, cell body plus dendrite) and ‘GCL’ are compared (*n = 5*). Data in (E) and (J) were represented as mean ± SEM and analysed using one-way ANOVA and Dunnett’s multiple comparisons test (ns *P* > 0.05, ***P < 0.001, ****P < 0.0001). Scale bar 100 µm, and see Supplementary Fig. 6 for separate channel images and Supplementary Table 2 for detailed statistical analysis.

P6 Ts65Dn has smaller cerebellum and fewer GCs in IGL than Eu.^44^^,^^53^ However, little is known about differences in PC morphology between Ts65Dn and Eu at P6. To examine the causes of the *t_d_*-dependent dMRI changes in ROI1 and ROI2 of P6 Ts65Dn, sections of the cerebellum were immunostained with anti-Calbindin, showing that the dendritic arborization of the PCs was significantly under-developed in Ts65Dn compared to Eu, i.e. PC proximal dendrites were much shorter in Ts65Dn (Fig. 4, c1 and d1). Postmitotic GCs in the IGL were less densely packed in Ts65Dn (Fig. 4, c2 and d2). The linear measurements (the thickness) of PC (cell body plus developing dendrites), IGL, and ‘ML plus EGL’ of Ts65Dn were 68.3 ± 8.2%, 81.1 ± 7.5%, and 96 ± 5.4% of Eu, respectively (Fig. 4E). Together, the results from OG-dMRI and histological analysis of P6 cerebellum suggest that the delayed development of PCs in Ts65Dn contributes to the OG-ADC reduction in ROI2 (the outer part of IGL and PCL) and that the underdeveloped GCs in IGL in Ts65Dn leads to the reduced OG-ADC in both ROI1 (WM and the inner part of IGL) and ROI2.

### *t_d_*-dependent dMRI of the adult Ts65Dn cerebellum

**Figure 5:**
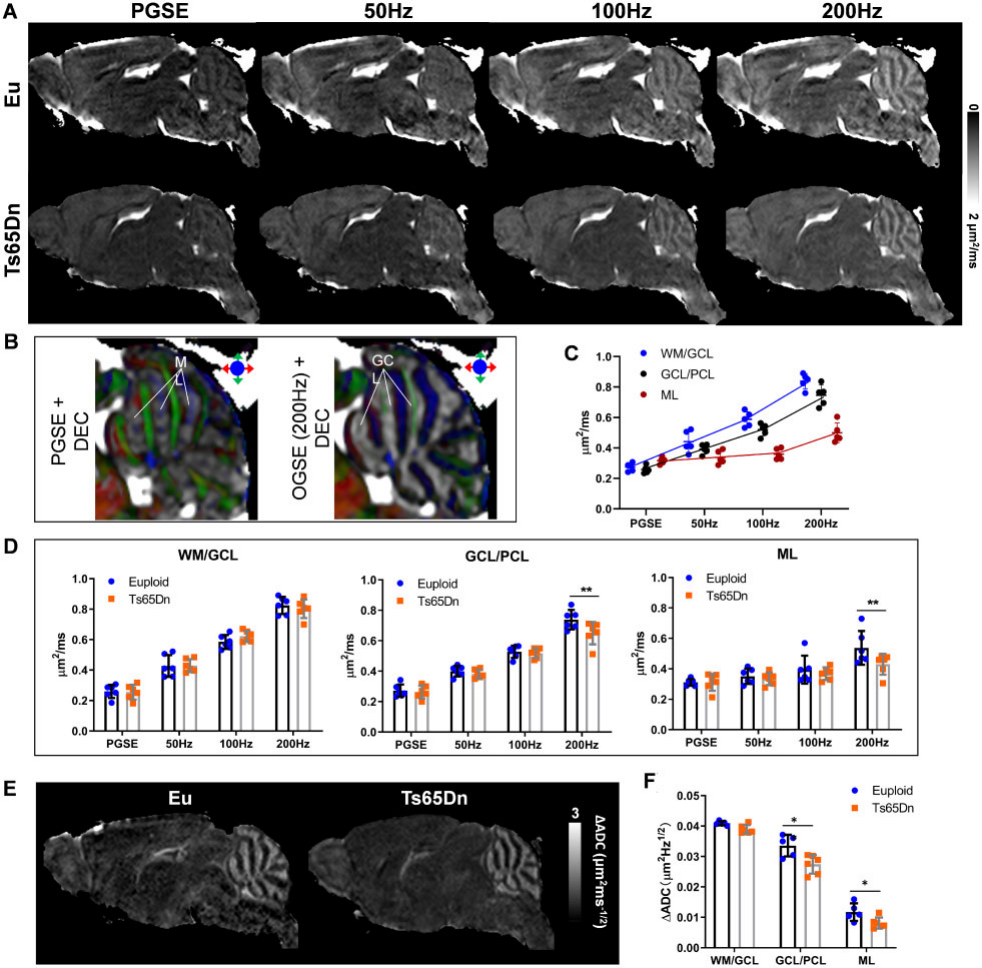
***T_d_*-dependent dMRI in the Eu and Ts65Dn mouse brains at P31.** (**A**) Mid-sagittal ADC maps of Eu and Ts65Dn mouse brains acquired with PG-dMRI and OG-dMRI at *f* of 50 Hz, 100 Hz and 200 Hz. (**B**) Overlay of the ADC maps on the DEC images to identify the spatial correspondence between the ADC contrasts and ROI definitions. The high ADC regions from PG-dMRI corresponded to the ML, and high ADC regions from OG-dMRI at 200 Hz corresponded to the GCL. (**C**) ADC measures in the ROI1 (WM/GCL), ROI2 (GCL/PCL) and ROI3 (ML) of the Eu brains from PG- and OG-dMRI in six P31 mice. (**D**) Comparison of ADC values at PG and OG-dMRI of 50–200 Hz between the Eu and Ts65Dn groups (*n *=* *6) in the three cerebellar ROIs. (**E**) Mid-sagittal ΔADC maps of the Eu and Ts65Dn mouse brains at P31. (**F**) Comparison of ΔADC between Eu and Ts65Dn groups in the three cerebellar ROIs. Data of (D) and (F) were analysed by two-way ANOVA and followed by *post hoc t*-test (*P <* *0.05 and **P <* *0.01), and see also Supplementary Table 2 for detailed statistical analysis.

At P31, the mouse cerebellum was fully developed. OG-ADC maps at *f* of 100–200 Hz exhibited a strong contrast in the cerebellum (Fig. 5A), with the high ADC region locating in the GCL and the low ADC part overlaying with the ML according to the spatial correspondence between the DEC and ADC maps (Fig. 5B). Interestingly, the ADC contrast was inverted in the PG-ADC map, with high ADC regions corresponding to ML, possibly due to the very restricted diffusion in the cellular layers and less restricted diffusion in the ML containing mainly the neurites for diffusion at long *t_d_*. The OG-ADC map at 50 Hz showed little contrast, possibly because it was at the transition of the contrast inversion from PG-ADC to OG-ADC at 100 Hz. The *t_d_*-dependency was much higher in the ROIs containing GCL than in the ML (Fig. 5C), which gave rise to the strong contrast between GCL and ML in the ΔADC map (Fig. 5E).

Ts65Dn had thinner a GCL than Eu based on the highlighted regions in the 100-200Hz OG-ADC maps and ΔADC map (Fig. 5A and E). The ADC reduction was only found to be significant at *f* of 200 Hz in both ROI2 (GCL/PCL) and ROI3 (ML) (*P* <* *0.01, Fig. 5D). At *f* of 200 Hz, the OG-ADC ratio of Ts65Dn to Eu were 85.6%±4.3% in GCL/PCL and 89.2 ± 7.7% in ML, which was comparable to the histological based quantification showing that the layer thickness ratio of Ts65Dn to Eu were 84.8 ± 4.8% in GCL and 87.2 ± 3.3% in PC [cell body plus mature dendrites (ML), Fig. 4J]. The ΔADC differences were also detectable in GCL/PCL (*P* =* *0.0003) and ML (*P* =* *0.022, Fig. 5F). Thus, our findings demonstrated that despite the subtler microstructural alteration in Ts65Dn cerebellum at adult than P6, the diffusion *t_d_*-dependence, as a marker, could reliably detect the remaining abnormalities.

## Discussion

In this study, we have investigated the use of *t_d_*-dependent dMRI to detect cerebellar pathology in a DS mouse model ‘Ts65Dn’ at neonatal and early adult stages. Our results demonstrate that short-*t_d_* diffusion with OG-dMRI is more sensitive than PG-dMRI to the altered cerebellar microstructures in Ts65Dn. The advantage of OG-dMRI begins at a relatively low *f* of 50 Hz and becomes more pronounced as *f* increases (or *t_d_* decreases). The capability of *t_d_*-dependent dMRI to resolve microstructures largely depends on the attainable *t_d_* or *f*. Although high *f* is currently only attainable with high gradient strength on preclinical systems, it is encouraging to see that OG-dMRI at 50 Hz could significantly improve the detection sensitivity in the developing cerebellum, which is feasible on clinical scanners.^54–57^

Based on the spatial correspondence between the DTI contrast and cerebellar laminar structures illustrated in this study, our results demonstrate that (i) the diffusivity difference between Ts65Dn and Eu is most evident in the IGL/PCL at P6, and the detection sensitivity increases with oscillating frequency *f*; (ii) the microstructural differences are subtler at P31 than P6 with remaining alterations in the GCL/PCL and ML, which is only detectable at *f* of 200 Hz; and (iii) the high *t_d_*-dependence in the normal cerebellum is reduced in the Ts65Dn mice. OG-ADC maps of P6 Ts65Dn (Fig. 3) agree well with the histological analysis showing that its PC size and IGL thickness are ∼ 68% and ∼81% of Eu (Fig. 4A–E). At P31, the differences between Ts65Dn and Eu groups become less evident, but OG-ADC at *f* of 200 Hz reliably detects the structural deficits and matches well with the corresponding histological analysis where the thickness of trisomic GCL and ML are reduced to ∼85% and 87% of Eu (Fig. 4F–J), in line with previous findings that GCL and ML of Ts65Dn are reduced to 87% and 92% of Eu at adult.^42^^,^^44^

**Figure 6:**
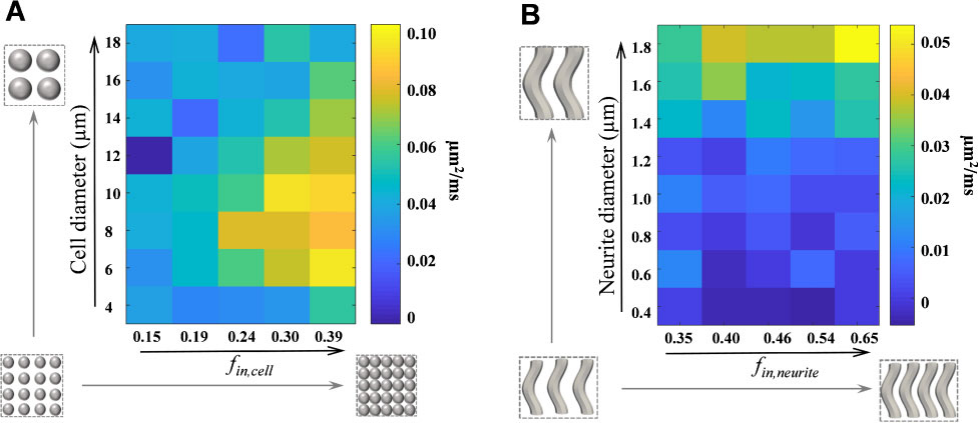
**Simulation of ΔADC in response to microstructural changes in cellular and neurite phantoms.** (**A**) ΔADC fitted from simulated PG- and OG-dMRI data at 20 ms, 50 Hz, 100 Hz and 200 Hz, based on a cell phantom with diameters varying from 4 to 18 µm and intracellular fraction varying from 0.15 to 0.39 for each diameter. (**B**) ΔADC fitted from simulated PG- and OG-dMRI data at 20 ms, 50 Hz, 100 Hz and 200 Hz, based on a cylinder phantom with diameters varying from 0.4 to 1.8 µm and intracellular fraction varying from 0.35 to 0.65 for each diameter.

The diffusion *t_d_*-dependence (ΔADC) is linked to several microstructural features.^19^^,^^58^ Our simulation results (Fig. 6) show that for the cell phantoms, ΔADC increases as intracellular fraction increases from 0.06 to 0.39; while the relationship between ΔADC and cell diameter is nonlinear, with the maximum ΔADC around 8–10 µm. For the neurite phantoms, ΔADC increases with neurite diameter from 0.4 to 1.8 µm, and it also increases with intra-neurite fraction at the same time. The results suggest that the increase of ΔADC could be attributed to the increase of cellular and/or neurite densities and that the reduced ΔADC in Ts65Dn is likely associated with the reduced GC density^31^^,^^44^ as well as the reduced neurite density such as the abnormal arborization of the PC dendrites^43^^,^^59^^,^^60^ and/or the reduced number of PCs.^61^

Besides the new dMRI method for the cerebellar microstructure analysis, to the best of our knowledge, this study has another two important phenotypic findings, which could be applied in the regular DS research labs that are working on cerebellar hypoplasia and its therapy. (i) It is the first time that we have the direct PC morphology comparison between Eu and Ts65Dn in the postnatally developing cerebellum (P6) and show that P6 Ts65Dn has a very significant development delay in PCs detected by histology (Fig. 4), while almost all previous studies about trisomic PCs are in adult cerebellum. PCs secrete sonic hedgehog, a potent morphogen, to induce GC precursor proliferation to produce more GCs during the first two postnatal weeks, arguably the most important window for cerebellar development.^28^^,^^51^^,^^62^ Delays in PCs development likely directly affect sonic hedgehog production in the cerebellum and thus affect GC precursors proliferation and GC number, which could be an important cause for DS cerebellar hypoplasia. (ii) This is the first time that we have unbiasedly measured volumetric changes of trisomic brain and cerebellum during the postnatal development (P6) and directly compared them with that of the adulthood (P31) (Table 1), as previous studies on brain morphometry of DS mouse models by MRI are in adults only. Brain and cerebellum volume reductions in Ts65Dn are much more significant at P6 than P31 based on volumetric MRI: The reduction in the cerebellar volume is 34% in P6 Ts65Dn versus 14% in P31 Ts65Dn; and the reduction in the whole-brain volume is 14% in P6 Ts65Dn versus 7% in P31 Ts65Dn. Together, those two robust P6 cerebellar phenotypes, PC morphology and cerebellar volume, could reliably evaluate the efficacy of the prenatal and perinatal cerebellum therapies.

For the basic and translational DS research, animal models that are evolutionarily more closed to humans, richer in the number of HSA21 orthologs, more accessible and sensitive for physiological and behavioural tests, and more ‘Pharma-preferred’ for drug testing are the better. The four main genetic criteria for evaluating a DS mouse model are aneuploidy (an extra freely segregating chromosome), mosaicism, the number of functionally trisomic HSA21 genes/orthologs, and the number of functionally trisomic or monosomic non-HSA21 genes/orthologs.^63^^,^^64^ Although Ts65Dn mouse has been transforming DS research for over 25 years, it could be improved by increasing the number of trisomic HSA21 orthologs and reducing the number of trisomic non-HSA21 orthologs.^40^^,^^65^^,^^66^ More than 20 trisomic models for various subsets of HSA21 genes/orthologs have been developed since, but certain critical drawbacks limit broader applications. Dp(16)1Yey and the ‘triple’ mouse, Dp(10)1Yey/+; Dp(16)1Yey/+; Dp(17)1Yey/+, have more trisomic HSA21 orthologs but are not aneuploidy,^67^^,^^68^ while Tc1 is trisomic for ∼75% of 213 HSA21q protein-coding genes but has the mosaicism issue.^69^ Until recently, the most complete genetic mouse model of DS extant, TcMAC21, contains a freely segregating hybrid HSA21 chromosome, the 34 MB long arm of HSA21 (HSA21q) engineered with a mouse artificial chromosome.^63^ TcMAC21 is not mosaic and trisomic for 199 (93%) HSA21q protein-coding genes, and it is not trisomic for any non-HSA21 genes/orthologs. Among aneuploid mouse models of DS (Ts65Dn, Tc1 and TcMAC21) that have an extra freely segregating chromosome of HSA21 genes/orthologs, the disproportionally small cerebellum is consistently reported based on the unbiased MRI,^42^^,^^63^^,^^70^ and other few repeatable and potentially translatable phenotypes include deficits in Morris water maze (MWM) tests and hippocampus-LTP. As DS is a human condition, it is debatable that mouse models with HSA21 are better or mouse models with HSA21 mouse orthologs are better. Thus, the combination usage of humanized (TcMAC21) and non-humanized (Ts65Dn or its upgrade) DS mouse models are recommended for DS research, particularly for a drug screen. In the future, *t_d_*-dependent dMRI will be applied for studying microstructural alteration in the TcMAC21 cerebellum.

Other than creating better genetic models of trisomy 21, it is also critical to standardize and upgrade methods for analysing DS phenotypes. A recent study^71^ suggests that phenotypic drift in neuroanatomical and behavioural analyses exist in Ts65Dn after comparing different cohorts from two Ts65Dn strains, Ts65Dn^1924^ (C57BL/6JEiJ × C3Sn with mutated Pde6b, Jax 001924) and Ts65Dn^5252^ (C57BL/6JEiJ × C3Sn with wildtype Pde6b, Jax 005252). Although many prenatal and postnatal phenotypes are not stable, the most critical phenotype—‘learning and memory deficits in MWM’ of the current cohort ‘5252^Cryo2010^’, is still consistent with the original MWM finding^40^ and their earlier MWM finding,^72^ and it also should be pointed out that the design of MWM is strongly correlated with the sensitivity. To analyse the size/volume of the brain and cerebellum that are related to DS phenotypes, unbiased 3D-MRI should be listed as a standard method for DS research^42^^,^^51^^,^^63^^,^^70^ as two-dimensional histology could be biased and/or create significant variation. For analysing neuronal or non-neuronal cell density in different brain structures of DS mouse models, particularly, small and densely packed cell types such as in DG of hippocampus and GCL of cerebellum, unbiased stereology of complete histological sections labelled with genetic markers or H&E staining should be recommended.^44^^,^^73^ Using immunostaining of one or a few sections with antibodies such as anti-NeuN to quantify GC density is challenging and likely produces inconsistent data, as results are significantly influenced by the slice thickness and location, antibody quality and penetration, and sampling/counting methods. The dMRI based techniques can capture the microscopic organization based on behaviours of water diffusion and thus provide pathological information in addition to the volumetric measures. Particularly, the unique sensitivity of short-*t_d_* diffusion is especially useful for characterizing GC density in the cerebellum, which are among the smallest neurons in the brain with a diameter of 5–6 µm and a large nuclear-to-cell ratio. Given $l=\sqrt{2Dt_{d}}$ and *D* of 2 µm^2^/ms, the diffusion distance of water molecules is approximately 4.5, 3.2 and 2.2 µm for *f* of 50, 100 and 200 Hz, which is on the order of GCs (for 50 Hz) or subcellular structures and dendritic processes (for 100–200Hz). Therefore, diffusion at short *t_d_* is well-suited to probe the cellular and neurite alterations in DS. Moreover, the combination of the microstructural pathology with DTI-based connectivity^74^ and anatomical MRI-based morphological features^75^^,^^76^ would likely provide systematic information on cerebellar disorders and potential therapeutic effects in a non-invasive way, and these approaches can be conveniently translated to clinical practice.

There are several limitations in the current study. Our experiments are performed in fixed brain specimens using a 3D sequence, which provides superior resolution for structural delineation compared to conventional two-dimensional multislice sequences. However, death and chemical fixation can alter tissue microstructural properties^53^^,^^77^ and introduce changes in ADC measurements. We previously have shown that *t_d_*-dependence increased in the fixed brain, and the ADC difference between *in vivo* and *ex vivo* brains is narrowed at high *f*.^78^ How this may change the sensitivity of *t_d_*-dependent dMRI to pathology needs to be further investigated. Furthermore, since our scan protocol only includes a single b-value of 1 ms/µm^2^, we are not able to employ complex biophysical models^15^^,^^17^ to reconstruct microstructural properties such as cell size and intracellular fraction that may improve quantification of the microstructural alteration. The limited number of oscillating frequencies (*f *=* *0, 50, 100, 200 Hz) also challenges the robustness of power-law fitting. Finally, although this study demonstrated the advantage of OG-dMRI in detecting cerebellar abnormalities at a clinically achievable frequency of 50 Hz, the clinical value of short-*t_d_* diffusion requires further investigation due to challenges in imaging resolution, diffusion gradient and scan time limitation. Nevertheless, clinical translation of OG-dMRI is emerging.^79–82^ Advances in hardware, particularly, high-performance head-only gradient coils,^83–85^ make moderate oscillating frequencies (up to 100 Hz) feasible for human applications,^86^ and the improvement in pulse sequence and gradient design^29^^,^^87^^,^^88^ could further facilitate the clinical translation of OG-dMRI.

## Supplementary material

Supplementary material is available at *Brain Communications* online.

## Funding

This work is supported by the Ministry of Science and Technology of the People's Republic of China (2018YFE0114600 to D.W.), National Natural Science Foundation of China (61801424 and 81971606 to D.W.; and 61801421 and 81971605 to Y.Z.), Leading Innovation and Entrepreneurship Team of Zhejiang Province (202006140 to D.W.), and Eunice Kennedy Shriver National Institute of Child Health and Human Development (R01HD038384 to F.J.G. and R.H.R.).

## Competing interests

The co-author Susumu Mori is an employee of AnatomyWorks Inc.

## Supplementary Material

fcab062_Supplementary_Data

## Abbreviations

ADC =: apparent diffusivity coefficient
DEC =: directional encoded colourmap
dMRI =: diffusion MRI
DS =: Down syndrome
DTI =: diffusion tensor imaging
EGL =: external granular layer
Eu =: euploid
GC =: granule cell
GCL =: granule cell layer
HSA21 =: human chromosome 21
IGL =: internal granular layer
ML =: molecular layer
MWM =: Morris water maze
OG =: oscillating gradient
P =: postnatal day
PC =: Purkinje cell
PCL =: Purkinje cell layer
PFA =: paraformaldehyde
PG =: pulsed gradient
ROI =: regions of interest
td =: diffusion-time
WM =: white matter
