# Supplementary material for: Time-dependent diffusion MRI probes cerebellar microstructural alterations in a mouse model of Down syndrome
Source: Brain Commun. 2021 Apr 5;3(2):fcab062. doi: 10.1093/braincomms/fcab062 (PMC8063586; doi:10.1093/braincomms/fcab062)
Supplement: fcab062_Supplementary_Data [file fcab062_supplementary_data.zip › Supplementary_Tables_revised2.docx]

**Supplementary Tables**

Supplementary Table 1 - ICD10 codes for the specific causes of deaths

| Causes of deaths | ICD10-codes |
| --- | --- |
| Ischemic heart disease  Other cardiovascular disease | I20-I25  I10-I15, I27, I30-I52, I28, I70-I79 |
| Cerebro-vascular disease | I60-I69 |
| Pneumonia  Other respiratory disease | J13-J18  J00-J06, J10-J11, J20-J22, J30-J47, J60-J99 |
| Cancer | C00-C34, C37-C97, D00-D09 |
| Digestive disease  Urinary and genital disease  Endocrine disease | K00-K31, K70-K83, K35-67, K85-K93  N00-N08, N20-N23, N10-N19, N25-N99  E00-E07, E10-E90 |
| Mental disease | F00-F99 |
| Ill-defined conditions/senility/unknown cause  Other | R00-R99  A00-A09, A20-A99, B00-B90, B99,A15-19, D10-D48, D50-89, G00-G99, H00-H95, I00-I09, I26, I80-I99, O00-O99, L00-L99, M00-M99, Q20-Q28, Q00-Q18, Q30-Q99, P00-P86, X60-X84, Y87.0, X85-Y36 |

Supplementary Table 2 - ICD10 codes for the specific causes of hospitalisations

| Diagnoses | ICD10-codes |
| --- | --- |
| Infectious and parasitic diseases | A00-A64, B90, A65-A99, B00-B89, B91-B99 |
| Malignant neoplam | C16, C17-C18, C19-C21, C25, C15, C22-C24, C26, C33-C34, C50, C53-C55, C51-C52, C56-C58, C60-C63, C64-C68, C00-C14, C30-C32, C37-C49, C69-C80, C97, D00-D09, C81-C96, D25, D26-D27, D10-D24, D28-D48 |
| Nutritional and metabolic diseases | E05, E00-E04, E06-E07, E10-E14, E15-E16, E20-E90 |
| Diseases of blood and bloodforming organs | D50-D89 |
| Mental disorders | F00-F39, F40-F99 |
| Diseases of the nervous system and sensory organs | G40-G41, G00-G37, G43-G99, H00-H01, H10, H15-H16, H20, H30, H46, H02-H06, H11-H13, H17-H19, H21-H28, H31-H45, H47-H59, H65-H67, H70, H60-H62, H68-H69, H71-H75, H80-H95 |
| Diseases of the circulatory system | I00-I09, I10-I15, I21-I24, I20, I25, I44-I50, I30-I43, I51-I52, I60-I69, I70, I83-I84, I26-I28, I71-I82, I85-I99 |
| Diseases of the respiratory organs | J00-J06, J10-J11, J12-J18, J20-J22, J40-J46, J35, J30-J34, J36-J39, J47-J99 |
| Diseases of the digestive system | K00-K14, K25-K28, K20-K23, K29-K31, K35-K38, K40-K46, K56, K50-K52, K57-K59, K55, K60-K67, K90-K93, K80-K83, K70-K77, K85-K87 |
| Diseases of the genito-urinary system | N10-N12, N30, N20-N23, N00-N08, N13-N19, N25-N29, N31-N39, N991, N995-N999, N40, N41-N51, N60-N64, N70, N73, N83, N81, N91-N94, N950, N951-N959, N71-N72, N74-N80, |
| Diseases of the skin and subcutaneous tissue | L00-L08, L10-L99 |
| Diseases of the musculoskeletal system and connective tissue | M05-M09, M10-M14, M45, M15-M19, M43, M47-M49, M00-M03, M42, M46, M80-M94, M50-M54, M20-M41, M60-M79, M95-M99 |
| Symptoms, signs and ill-defined conditions | R00-R99, N994, Z00-Z13 |

Excluded are deliveries and complications of pregnancy and childbirth, congenital malformations and abnormalities, certain conditions originating in the perinatal period, injuries, and poisoning.

Supplementary Table 3 – Distribution of time to pregnancy (TTP) among people in the Omnibus (born 1953-1976) who became pregnant, stopped trying and were still trying at interview in 1994

|  | Women |  |  | Men |  |  |
| --- | --- | --- | --- | --- | --- | --- |
| TTP, months | Became pregnant | Still trying | Gave up trying | Became pregnant | Still trying | Gave up trying |
| <2 months | 2,991 (53.6) | 45 (10.7) | 40 (18.7) | 2,452 (58.9) | 37 (10.3) | 57 (31.8) |
| 2-9 months | 1,680 (30.1) | 82 (19.5) | 42 (19.6) | 1,162 (27.9) | 80 (22.2) | 36 (20.1) |
| 10-17 months | 326 (5.8) | 66 (15.7) | 25 (11.7) | 199 (4.8) | 31 (8.6) | 18 (10.1) |
| ≥18 months | 581 (10.4) | 228 (54.2) | 107 (50.0) | 351 (8.4) | 212 (58.9) | 68 (38.0) |
| Missing | 9,674 (63.4) | 14,831 (97.2) | 15,038 (98.6) | 10,017 (70.6) | 13,821 (97.5) | 14,002 (98.7) |

All estimates are numbers (percentages).

Supplementary Table 4 - Distribution of time to pregnancy (TTP) in the total study population of Danish women and men born 1931-1976

|  | Women |  |  | Men |  |  |
| --- | --- | --- | --- | --- | --- | --- |
| TTP, months | MADT | Omnibus | Combined | MADT | Omnibus | Combined |
| Initial sample |  |  |  |  |  |  |
| <2 months | 108 (76.6) | 136 (63.9) | 244 (68.9) | 99 (79.8) | 37 (62.7) | 136 (74.3) |
| 2-9 months | 21 (14.9) | 50 (23.5) | 71 (20.1) | 17 (13.7) | 15 (62.4) | 32 (17.5) |
| 10-17 months | 5 (3.6) | 8 (3.8) | 13 (3.7) | 6 (4.8) | 1 (1.7) | 7 (3.8) |
| ≥18 months | 7 (5.0) | 19 (8.9) | 26 (7.3) | 2 (1.6) | 6 (10.2) | 8 (4.4) |
| Total numbers | 141 (7.9) | 213 (3.5) | 354 (4.5) | 124 (7.1) | 59 (1.3) | 183 (2.9) |

All estimates are numbers (percentages).

Supplementary Table 5 - Hazard ratios (HRs) and Incidence Rate ratios (IRRs) for mortality and hospitalisations among Danish women and men born 1931-1976 with a time to pregnancy (TTP) of 2-9, 10-17 and 18 months or more relative to those with a TTP of less than 2 months, unadjusted, with age as the underlying time scale, for mortality and adjusted only for age at interview for hospitalisations

|  | Women |  |  | Men |  |  |
| --- | --- | --- | --- | --- | --- | --- |
| TTP, months | MADT | Omnibus | Combined | MADT | Omnibus | Combined |
| Mortality | HR (95% CI) | HR (95% CI) | HR (95% CI) | HR (95% CI) | HR (95% CI) | HR (95% CI) |
| <2 | 1 | 1 | 1 | 1 | 1 | 1 |
| 2-9 | 1.18 (0.93, 1.49) | 0.80 (0.58, 1.10) | 1.03 (0.86, 1.24) | 1.07 (0.87, 1.32) | 1.05 (0.77, 1.44) | 1.07 (0.90, 1.27) |
| 10-17 | 1.18 (0.77, 1.81) | 1.17 (0.71, 1.92) | 1.18 (0.85, 1.63) | 1.24 (0.89, 1.73) | 1.44 (0.84, 2.46) | 1.29 (0.97, 1.71) |
| ≥18 | 1.40 (1.01, 1.95) | 1.47 (1.06, 2.05) | 1.44 (1.14, 1.82) ^a^ | 1.21 (0.89, 1.66) | 1.29 (0.89, 1.85) | 1.24 (0.98, 1.57) |
| p-values for trend | 0.200 | 0.015 | 0.016 | 0.462 | 0.378 | 0.152 |
| Hospitalisation | IRR (95% CI) | IRR (95% CI) | IRR (95% CI) | IRR (95% CI) | IRR (95% CI) | IRR (95% CI) |
| <2 | 1 | 1 | 1 | 1 | 1 | 1 |
| 2-9 | 1.02 (0.85, 1.22) | 0.95 (0.83, 1.08) | 0.97 (0.87, 1.08) | 0.99 (0.84, 1.18) | 1.24 (1.04, 1.47) ^a^ | 1.16 (1.02, 1.32) |
| 10-17 | 1.01 (0.74, 1.37) | 0.95 (0.77, 1.18) | 0.96 (0.80, 1.15) | 0.92 (0.70, 1.22) | 1.08 (0.80, 1.46) | 1.03 (0.83, 1.28) |
| ≥18 | 1.32 (1.00, 1.74) | 1.20 (1.02, 1.41) | 1.22 (1.06, 1.40) ^a^ | 0.90 (0.72, 1.14) | 1.31 (1.09, 1.58) ^a^ | 1.20 (1.03, 1.40) |
| p-values for trend | 0.261 | 0.041 | 0.010 | 0.544 | 0.043 | 0.126 |

^a^Significant after adjusting for multiple testing.

HR: Hazard ratio, IRR: Incidence rate ratio, CI: confidence interval.

Supplementary Table 6 - Hazard ratios (HRs) and Incidence Rate ratios (IRRs) for mortality and hospitalisations among Danish women and men born 1931-1976 with a time to pregnancy (TTP) of 2-9, 10-17 and 18 months or more relative to those with a TTP of less than 2 months, including individuals who reported becoming pregnant despite the use of contraception in the lowest TTP group (<2 months), also those who were excluded from the main analysis due to missing TTP

|  | Women |  |  | Men |  |  |
| --- | --- | --- | --- | --- | --- | --- |
| TTP, months | MADT | Omnibus | Combined | MADT | Omnibus | Combined |
| Mortality | HR (95% CI) | HR (95% CI) | HR (95% CI) | HR (95% CI) | HR (95% CI) | HR (95% CI) |
| <2 | 1 | 1 | 1 | 1 | 1 | 1 |
| 2-9 | 1.18 (0.93, 1.49) | 0.88 (0.63, 1.23) | 1.07 (0.88, 1.29) | 1.00 (0.81, 1.24) | 1.08 (0.79, 1.49) | 1.04 (0.87, 1.24) |
| 10-17 | 1.02 (0.66, 1.56) | 1.15 (0.67, 1.98) | 1.07 (0.76, 1.50) | 1.16 (0.83, 1.63) | 1.49 (0.86, 2.57) | 1.27 (0.95, 1.69) |
| ≥18 | 1.39 (0.99, 1.95) | 1.46 (1.03, 2.06) | 1.40 (1.10, 1.80) ^a^ | 1.15 (0.82, 1.61) | 1.28 (0.87, 1.86) | 1.20 (0.94, 1.54) |
| p-values for trend | 0.229 | 0.086 | 0.063 | 0.725 | 0.397 | 0.258 |
| Hospitalisation | IRR (95% CI) | IRR (95% CI) | IRR (95% CI) | IRR (95% CI) | IRR (95% CI) | IRR (95% CI) |
| <2 | 1 | 1 | 1 | 1 | 1 | 1 |
| 2-9 | 1.07 (0.90, 1.27) | 0.93 (0.81, 1.06) | 0.97 (0.87, 1.08) | 0.98 (0.83, 1.16) | 1.18 (1.01, 1.39) | 1.13 (1.00, 1.29) |
| 10-17 | 1.13 (0.79, 1.61) | 0.88 (0.73, 1.07) | 0.94 (0.79, 1.11) | 0.93 (0.69, 1.24) | 1.05 (0.79, 1.40) | 1.03 (0.83, 1.27) |
| ≥18 | 1.47 (1.11, 1.95) ^a^ | 1.14 (0.96, 1.37) | 1.20 (1.03, 1.40) | 0.84 (0.67, 1.07) | 1.25 (1.04, 1.50) | 1.15 (0.98, 1.33) |
| p-values for trend | 0.064 | 0.053 | 0.023 | 0.655 | 0.043 | 0.126 |

^a^Adjusted for birth cohort, age at first attempt to become pregnant, years in school, smoking and BMI.

^b^Significant after adjusting for multiple testing.

HR: Hazard ratio, IRR: Incidence rate ratio, CI: confidence interval.

Number of observations for TTP <2 months: Women (MADT: 158, Omnibus: 730; Combined: 888). Men (MADT: 134, Omnibus: 359; Combined: 493).

Supplementary Table 7 – Hazard ratios (HRs) for mortality among Danish women and men in the Omnibus (born 1953-1976) with a time to pregnancy (TTP) of 2-9, 10-17 and 18 months or more relative to those with a TTP of less than 2 months, censored at age 45 years

|  | Women | Men |
| --- | --- | --- |
| TTP, months | Omnibus | Omnibus |
| Mortality | HR (95% CI) | HR (95% CI) |
| <2 | 1 | 1 |
| 2-9 | 1.03 (0.53, 2.00) | 1.52 (0.79, 1.93) |
| 10-17 | 1.89 (0.77, 4.65) | 1.31 (0.38, 4.46) |
| ≥18 | 2.18 (1.14, 4.17) | 0.59 (0.17, 1.96) |
| p-values for trend | 0.070 | 0.401 |

All results are adjusted for birth cohort, age at first attempt to become pregnant, years in school, smoking and BMI.

HR: Hazard ratio, CI: confidence interval.

Supplementary Table 8 – Hazard ratios (HRs) and Incidence Rate ratios (IRRs) for mortality and hospitalisations among Danish women and men in the MADT (born 1931-1952) with a time to pregnancy (TTP) of 2-9, 10-17 and 18 months or more relative to those with a TTP of less than 2 months, further adjusted for diseases related to pregnancy

|  | Women | Men |
| --- | --- | --- |
| TTP, months | MADT | MADT |
| Mortality | HR (95% CI) | HR (95% CI) |
| <2 | 1 | 1 |
| 2-9 | 1.19 (0.94, 1.51) | 1.07 (0.86, 1.32) |
| 10-17 | 1.01 (0.65, 1.57) | 1.20 (0.86, 1.67) |
| ≥18 | 1.39 (0.99, 1.96) | 1.19 (0.86, 1.66) |
| p-values for trend | 0.207 | 0.599 |
| Hospitalisation | IRR (95% CI) | IRR (95% CI) |
| <2 | 1 | 1 |
| 2-9 | 1.07 (0.90, 1.28) | 0.99 (0.84, 1.18) |
| 10-17 | 1.10 (0.77, 1.56) | 0.91 (0.69, 1.22) |
| ≥18 | 1.42 (1.07, 1.89) ^a^ | 0.85 (0.67, 1.08) |
| p-values for trend | 0.120 | 0.523 |

All results are adjusted for birth cohort, age at first attempt to become pregnant, years in school, smoking, BMI and diseases related to pregnancy.

^a^Significant after adjusting for multiple testing.

HR: Hazard ratio, IRR: Incidence rate ratio, CI: confidence interval.

Supplementary Table 9 – All-cause mortality among Danish women and men born 1931-1976 who never tried to become pregnant and those with missing values on time to pregnancy (TTP) relative to people who tried to become pregnant (study population)

|  | Women | Men |
| --- | --- | --- |
|  | HR (95% CI) | HR (95% CI) |
| Tried to become pregnant | 1 | 1 |
| Never tried to become pregnant | 1.44 (0.96, 2.14) | 1.41 (0.96, 2.06) |
| Tried to become pregnant | 1 | 1 |
| Missing data on TTP | 0.83 (0.60, 1.15) | 0.89 (0.67, 1.18) |

All results are adjusted for birth cohort, age at first attempt to become pregnant, years in school, smoking and BMI.

HR: Hazard ratio, CI: Confidence interval.
