# Supplementary figures and images for: Time-dependent diffusion MRI probes cerebellar microstructural alterations in a mouse model of Down syndrome
Source: Brain Commun. 2021 Apr 5;3(2):fcab062. doi: 10.1093/braincomms/fcab062 (PMC8063586; doi:10.1093/braincomms/fcab062)

**Supplementary Figure 1 - Flow chart of the study population**

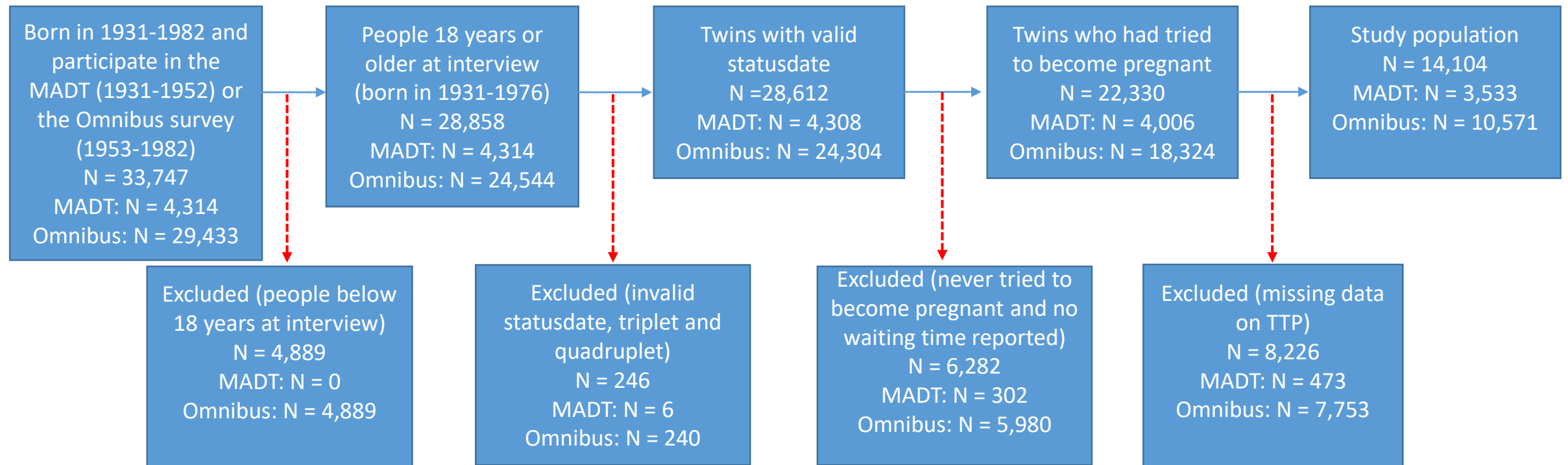

Supplement: fcab062_Supplementary_Data [file fcab062_supplementary_data.zip › Supp_Fig1.pdf]
